# High-Quality Chromosome-Level Genome Assembly of the Corsac Fox (*Vulpes corsac*) Reveals Adaptation to Semiarid and Harsh Environments

**DOI:** 10.3390/ijms24119599

**Published:** 2023-05-31

**Authors:** Zhihao Zhang, Tian Xia, Shengyang Zhou, Xiufeng Yang, Tianshu Lyu, Lidong Wang, Jiaohui Fang, Qi Wang, Huashan Dou, Honghai Zhang

**Affiliations:** 1 School of Life Science, Qufu Normal University, Qufu 273165, China; zhangzhihao1102@126.com (Z.Z.); qfxiatian1993@163.com (T.X.);; 2 Hulunbuir Academy of Inland Lakes in Northern Cold & Arid Areas, Hulunbuir 021000, China

**Keywords:** chromosome-level genome, Hi-C, semiarid adaption, *Vulpes corsac*, diet strategy

## Abstract

The Corsac fox (*Vulpes corsac*) is a species of fox distributed in the arid prairie regions of Central and Northern Asia, with distinct adaptations to dry environments. Here, we applied Oxford-Nanopore sequencing and a chromosome structure capture technique to assemble the first Corsac fox genome, which was then assembled into chromosome fragments. The genome assembly has a total length of 2.2 Gb with a contig N50 of 41.62 Mb and a scaffold N50 of 132.2 Mb over 18 pseudo-chromosomal scaffolds. The genome contained approximately 32.67% of repeat sequences. A total of 20,511 protein-coding genes were predicted, of which 88.9% were functionally annotated. Phylogenetic analyses indicated a close relation to the Red fox (*Vulpes vulpes*) with an estimated divergence time of ~3.7 million years ago (MYA). We performed separate enrichment analyses of species-unique genes, the expanded and contracted gene families, and positively selected genes. The results suggest an enrichment of pathways related to protein synthesis and response and an evolutionary mechanism by which cells respond to protein denaturation in response to heat stress. The enrichment of pathways related to lipid and glucose metabolism, potentially preventing stress from dehydration, and positive selection of genes related to vision, as well as stress responses in harsh environments, may reveal adaptive evolutionary mechanisms in the Corsac fox under harsh drought conditions. Additional detection of positive selection for genes associated with gustatory receptors may reveal a unique desert diet strategy for the species. This high-quality genome provides a valuable resource for studying mammalian drought adaptation and evolution in the genus *Vulpes*.

## 1. Introduction

The dry climate in the desert area of central Eurasia is mainly characterized by hot and dry summers, cold and dry winters, and the gradual desertification of natural landscapes [1]. Extreme temperatures, food deprivation, and excessive solar radiation present formidable challenges for local species [2]. However, a wide variety of organisms have evolved the ability to adapt to heat and drought stress. For example, camels exhibit adaptive features in fat and water metabolism, as well as in response to heat, strong UV light, and asphyxiating stress [3]. Maintenance of homeostasis under acute dehydration in cactus mice in the desert zone of North America and in livestock, such as desert sheep and Liangzhou donkeys, responds as an adaptation to hot drought [4,5,6]. Although such adaptive features have been described in several species, little is known about the genetic basis of the Corsac fox drought adaptation [7]. Therefore, we investigated the genetic basis of drought adaptation in Corsac foxes at the genomic level.

The Corsac fox belongs to the genus *Vulpes*, family Canidae, and order Carnivora and mainly inhabits steppe, desert, and semi-desert areas in northern and central Asia [8,9]. The Corsac fox is not listed in the CITES Appendices due to its large population size, although its survival is threatened by human activities, and its habitat is subject to degradation, which has attracted increasing attention from researchers [10]. The publication of transcriptome, mitochondrial genome, and metagenomic data on the Corsac fox has provided favorable support for niche adaptation and the evolutionary status of the species, although these investigations still lack the support of complete and high-quality genomic data [8,11,12,13].

Typical of its arid and semiarid habitat, the Corsac fox has evolved a set of adaptations to extreme environments [8]. Its behavioral and anatomical adaptations to complex temperature changes include nocturnal activity, hiding in burrows during the day to avoid solar radiation, larger ears to facilitate heat dissipation, and thicker fur to survive cold temperatures [10,14]. The width of the food niche of the Corsac fox varies seasonally [15]. Rodents are its primary food source, but the species also preys on birds and insects and even feeds on some plant seeds, ensuring a wide range of food sources [10,11,15,16,17]. The Corsac fox can also forgo food and water for extended periods, which helps in maintaining a low metabolism in response to sudden climatic changes [15]. It possesses very acute senses of sight, hearing, and smell, which help it in targeting its prey more precisely [10,11,15]. The harsh environment often puts the health of the fox at significant risk. Rabies and some parasites were reported in the Corsac fox, whose immune mechanisms require further exploration [18,19,20]. While the noted behaviors and physiological capacities can account for the adaptation of the Corsac fox to extreme environments, the underlying molecular mechanisms still require elucidation.

In this study, genomic DNA was extracted from muscle tissue samples of the Corsac fox, and a chromosome-scale representation of its genome was assembled. The aim of this study was to determine the unique adaptive characteristics of the Corsac fox in hot and arid environments. Therefore, we explored genes in the Corsac fox genome that may be associated with microenvironmental adaptation. This study provides a foundation to help unravel the molecular mechanisms of environmental adaptation in Corsac foxes and highlights the need for high-quality genomes to address speciation.

## 2. Results

### 2.1. Genome Sequencing and Assembly

A total of 132 Gb of reads (55.25×) with an insert size of 150 bp were obtained using the Illumina HiSeq 2500 platform, and a total of 282 Gb of reads (118.03×) were obtained with the Nanopore PromethION platform (Table S1). We also received ~268.65 Gb of Hi-C reads using the Illumina PE150 platform. We obtained ~682.65 Gb of raw genome sequence data for the genome assembly and other related analyses (Table S2). Additionally, 57.63 Gb of clean transcriptomic data were obtained for genome annotation (Table S3).

**Figure 1:**
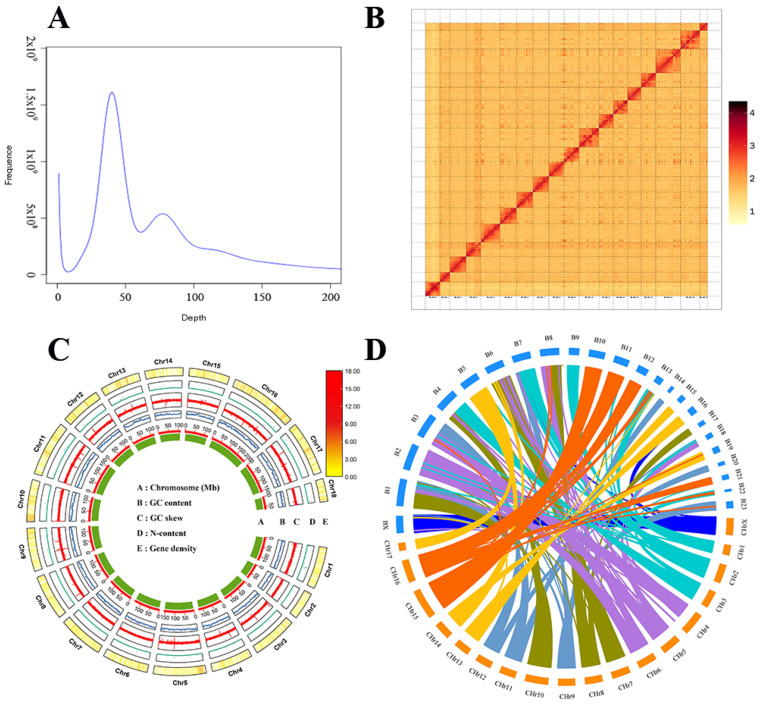
(**A**) Genome survey of *Vulpes corsac* using 17-mer analysis. K-mer depth on the horizontal axis and K-mer depth frequency on the vertical axis. (**B**) Genome-wide all-by-all Hi-C interaction identified 18 linkage groups. (**C**) Genome characteristics of *Vulpes corsac*. (**D**) The chromosome-scale synteny analysis between the Corsac fox and the Arctic fox genome, which was visualized using TBtools v2023.2.10 (. The Arctic fox chromosomes are represented by the numbered bands spanning the upper portion of the plot (B1–B23 and BX). The Corsac fox is represented by their respective chromosome number (CHr1–CHr17 and CHrX) spanning the lower half of the plot.

Before genome assembly, the Corsac fox genome estimated using 17-mer analyses was ~2.41 Gb (Table S4, Figure 1A). We also obtained a preliminary ~2.35 Gb genome assembly (contig N50: 47.65 Mb) using NextDenovo (Table S5). Using the Hi-C scaffolding method, the contigs in the initial group were anchored and oriented to the chromosomal scale of the community. After the Hi-C corrected contigs were placed in the ALLhic pipeline for segmentation, orientation, and sequencing, the final 99.49% of the assembled sequences were anchored to 18 pseudochromosomes with chromosome lengths ranging from 64.55 Mb to 202.44 Mb (Table S6, Figure 1B). This result is in agreement with the karyotyping results, which are based on cytological observations [21]. Following Hi-C correction, the final genome assembly was 2.2 Gb, scaffold N50 was 132.2 Mb, and contig N50 was 41.62 Mb (Table S7). The Circos plot is shown in Figure 1C.

To evaluate the completeness of the genome, we first performed BUSCO v2.3.1 analysis using the mammalia_odb10 database [22]. We found that 92.6% of the 9226 BUSCO genes in the genome were complete (Figure S1. The 99.68% read-mapping rate, 99.82% genome coverage rate, 0.106% heterozygous SNP rate, and 0.000118% homologous SNP rate of the final assembled genome served to verify its consistency and completeness. The GC (guanine–cytosine) concentration was approximately 41.24%, and the scatter plot indicated no significant separation of GC and no other exogenous contamination in the genome (Figure S2). The result conclusively indicates that the chromosomal genome assembly of the Corsac fox was of high quality and accuracy (Table S8).

We performed an interspecies synteny analysis of the assembled Corsac fox genome with that of the Arctic fox to assess the accuracy of the genome assembly [23]. There was a high degree of co-linearity between the two genomes, consistent with the close phylogenetic relationship between these species (Figure 1D).

### 2.2. Genome Annotation

The Repeat elements were annotated based on homology and De novo prediction. We first identified approximately ~767 Mb of repetitive elements in the Corsac fox genome, representing 32.67% of the total genome size (Table S9, Figure S3). Gene structure predictions were made using Augustus v3.2.3, Glimmer v3.0.4, SNAP v2013.11.29, GeneID v1.4, and GenScan v1.0 software, annotating *Ailuropoda melanoleuca*, *Canis familiaris*, *Homo sapiens*, *Mus musculus*, and *Vulpes vulpes* (Table S10, Figure S4). The annotated protein-coding genes in the *V. corsac* genome were identified using De novo prediction, homology-based prediction, and transcriptome sequencing-based methods. The above results were combined using the gene prediction integration software EVidenceModeler. A total of 22,501 protein-coding genes were annotated in the *V. corsac* genome (Table S11, Figure S5). Functional annotation revealed 20,511 genes, up to 91.2% of the *V. corsac* genes, with annotated functions in several databases, including TrEMBL, GO, KOG, SwissProt, InterPro, and NR (Table S12, Figure S6). Non-coding RNAs (ncRNAs) were annotated using various methods. Prognostic results showed that the ncRNAs in the *V. corsac* genome included 143,320 transfer RNAs (tRNAs). The ncRNAs in the Corsac genome included 16,395 tRNAs, 21,907 miRNAs, 481 ribosomal rRNAs, and 2448 snRNAs (Table S13). These results indicate that the quality of our annotated Corsac fox genome is reliable.

### 2.3. Unique Genes and Molecular Phylogenetic Analysis

The homologous relationships between the Corsac fox and the other assessed species were confirmed by aligning the protein sequences using OrthoFinder. A total of 20,070 gene families were identified in these species, 12 of which shared 13,379 core gene families. The Corsac fox genome contained 124 unique genes that were not shared by other species (Figure S7).

**Figure 2:**
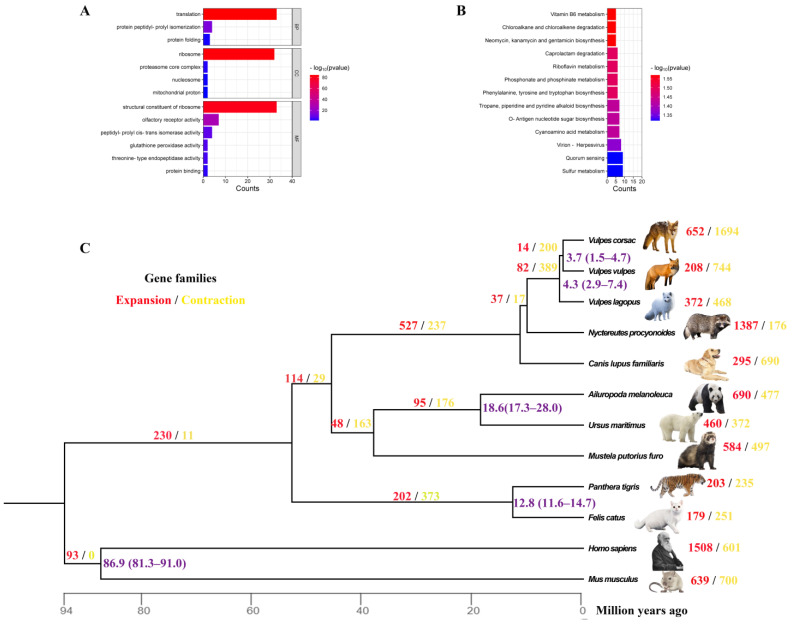
(**A**) GO classification of Unique genes of *V. corsac*, including 13 significant GO categories (*p* < 0.05). BP: Biological process; CC: Cellular component; MF: Molecular function. (**B**) Significant KEGG enrichment in Unique genes. We selected the top 13 KEGG terms ranked by count in distinctive genes. (**C**) Gene family evolution between genomes of *Vulpes corsac* and 11 other species, the orange number indicates gene family expansions, and red indicates gene family contractions. The length of the branch indicates the divergence time. MRCA: Most Recent Common Ancestor. Mya: million years ago.

Gene Ontology (GO) and Kyoto Encyclopedia of Genes and Genomes (KEGG) enrichment analyses in the species’ specific gene families were performed to identify their functions. The GO enrichment analysis showed that a large proportion of significantly enriched GO terms (*p* < 0.05) were closely related to protein reactions (Table S14, Figure 2A). An additional seven unique genes were significantly enriched in olfactory receptor activity. KEGG enrichment analysis of these specific genes showed that a large proportion of significantly enriched KEGG pathways (*p* < 0.05) were closely related to substance synthesis, including ko00400 (phenylalanine, tyrosine, and tryptophan biosynthesis, *p* = 0.03218), ko00524 (neomycin, kanamycin, and gentamicin biosynthesis, *p* = 0.026889), ko00541 (O-Antigen nucleotide sugar biosynthesis, *p* = 0.037442), ko00960 (tropane, piperidine, and pyridine alkaloid biosynthesis, *p* = 0.037442) and substance metabolism, including phosphonate and phosphinate metabolism, cyanoamino acid metabolism, riboflavin metabolism, vitamin B6 metabolism, and sulfur metabolism (Table S15, Figure 2B).

To explore the evolutionary status of the Corsac fox, we inferred its phylogenetic relationships with four species of canids, five other representative carnivores (Ursidae, Felidae, and Mustelidae), *Homo sapiens*, and *Mus musculus*. A phylogenetic tree was constructed using 12,620 single-copy orthologs of the Corsac fox and other mammals, which indicated that the Corsac fox clustered with the Red fox (*Vulpes vulpes*). The divergence time estimate indicates that the Corsac fox was separated from the closest species by approximately 3.7 MYA (Figure 2C).

### 2.4. Gene Family Analysis

It is possible that gene family expansion and contraction are one of the reasons for the adaptive differences between the investigated species [24]. This was present in all analyzed species to varying degrees. Of the three *Vulpes* genera, the Corsac fox had a greater number of gene family expansions and contractions than the Red and Arctic fox using CAFÉ5 [25]. There were 652 expansions and 1694 contractions in the Corsac fox, 208 expansions and 744 contractions in the Red fox, and 372 expansions and 468 contractions in the Arctic fox. We performed functional enrichment analyses of the gene families of these three species and found that the expanded gene families of the Corsac fox and Arctic fox were enriched for more pathways associated with energy metabolism and stress responses. Compared to the Red fox and Arctic fox, the Corsac fox had more pathways associated with protein responses in the expanded gene families. In the Red fox, more pathways related to energy metabolism and protein response were present in the contracted gene families. The gene families associated with olfactory receptor activity were contracted to varying degrees in the Corsac fox and Arctic fox gene families (Tables S16 and S17). We focus here more on the contracted and expanded gene families of the Corsac fox.

**Figure 3:**
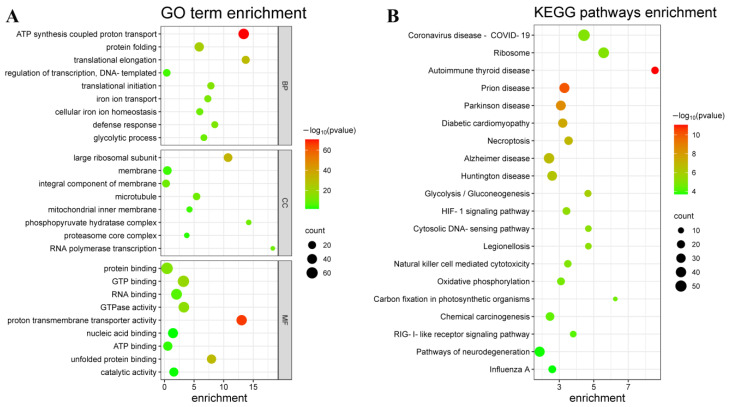
(**A**) GO classification of expanded gene families, including 26 significant GO categories (*p* < 0.05). BP: Biological process; CC: Cellular component; MF: Molecular function. (**B**) Significant KEGG enrichment in expanded gene families. We selected the top 20 KEGG terms ranked by count in expanded gene families.

Gene family expansion is often associated with the adaptive divergence of the species. Consequently, we obtained 261 gene families of development in the Corsac fox genome. Further functional enrichment analysis of the expanded gene families highlighted 120 significantly enriched GO terms (corrected *p* < 0.05) and 47 KEGG pathways (corrected *p* < 0.05) (Tables S18 and S19). GO enrichment analysis of these expanded gene families showed that a large proportion of significantly enriched GO terms were closely related to protein reaction and binding, including GO:0006468 (protein phosphorylation, *p* = 1.29 × 10^−6^), GO:0031369 (translation initiation factor binding, *p* = 0.004663), GO:0018101 (protein citrullination, *p* = 0.004662783), GO:0018095 (protein polyglutamylation, *p* = 6.65 × 10^−5^), GO:0005852 (eukaryotic translation initiation factor 3 complex, *p* = 0.0297035), GO:0051603 (proteolysis involved in cellular protein catabolic process, *p* = 0.0025696), GO:0006413 (translational initiation, *p* = 2.71 × 10^−14^), GO:0006355 (regulation of transcription, DNA-templated, *p* = 3.98 × 10^−5^), GO:0005515 (protein binding, *p* = 7.22 × 10^−14^). Four significantly enriched pathways were found to be related to energy metabolism, consisting of GO:0006096 (glycolytic process, *p* = 6.58 × 10^−10^), GO:0019752 (carboxylic acid metabolic process, *p* = 1.78 × 10^−7^), GO:1990544 (mitochondrial ATP transmembrane transport, *p* = 4.32 × 10^−7^), and GO:0006006 (glucose metabolic process, *p* = 2.07 × 10^−5^), and nine significantly enriched pathways were found to be related to stress response, including GO:0006955 (immune response, *p* = 0.0468476), GO:0006303 (double-strand break repair via nonhomologous end joining, *p* = 0.004662783), GO:0004129 (cytochrome-c oxidase activity, *p* = 0.000813394), GO:0016209 (antioxidant activity, *p* = 9.66 × 10^−8^), GO:0031072 (heat shock protein binding, *p* = 5.16 × 10^−13^), GO:0006952 (defense response, *p* = 2.46 × 10^−13^), GO:0016616 (oxidoreductase activity, acting on the CH-OH group of donors, NAD or NADP as acceptor, *p* = 6.35 × 10^−9^), GO:0016620 (oxidoreductase activity, acting on the aldehyde or oxo group of donors, NAD or NADP as acceptor, *p* = 4.41 × 10^−9^), and GO:0016491 (oxidoreductase activity, *p* = 0.00044571). Six significantly enriched pathways were related to iron ion reactions, consisting of GO:0006826 (iron ion transport, *p* = 1.48 × 10^−12^), GO:0006879 (cellular iron ion homeostasis, *p* = 2.71 × 10^−10^), GO:0008199 (ferric iron binding, *p* =1.48 × 10^−12^), GO:0051536 (iron-sulfur cluster binding, *p* = 0.002152932), GO:0051537 (two iron, two sulfur cluster binding, *p* = 0.006281612), and GO:0016226 (iron-sulfur cluster assembly, *p* = 0.044566349) (Figure 3A). Among the 47 significantly enriched KEGG pathways, eight were closely related to energy metabolism, stress response, and immune diseases (Figure 3B).

Contracted gene families may lead to morphological, physiological, and metabolic adaptations in species, thus revealing the possible molecular and cellular mechanisms behind these adaptive phenotypes [26]. In the enrichment analysis, we also found that 219 contracted gene families in the Corsac fox genome were significantly associated with 34 GO terms (corrected p < 0.05) and 47 KEGG pathways (corrected *p* < 0.05) (Tables S20 and S21). Enrichment analysis of these contracted gene families revealed that a large proportion of significantly enriched pathways were associated with substance metabolism, including ko00053 (Ascorbate and aldarate metabolism, *p* = 1.18 × 10^−5^), ko00140 (Steroid hormone biosynthesis, *p* = 5.34 × 10^−4^), ko00524 (Neomycin, kanamycin, and gentamicin biosynthesis, *p* = 4.86 × 10^−2^), ko00625 (Chloroalkane and chloroalkene degradation, *p* = 4.86 × 10^−2^), ko00750 (Vitamin B6 metabolism, *p* = 4.86 × 10^−2^), ko00790 (Folate biosynthesis, *p* = 2.53 × 10^−2^), ko00830 (Retinol metabolism, *p* = 4.33 × 10^−4^), etc. Functional pathways associated with olfactory receptor activity (GO:0004984, *p* = 3.76 × 10^−61^), olfactory transduction (ko04740, *p* = 8.98472 × 10^−8^), and phototransduction (ko04744, *p* = 0.023427737) were also enriched in the contracted gene families.

### 2.5. Positive Selection of Genes

**Figure 4:**
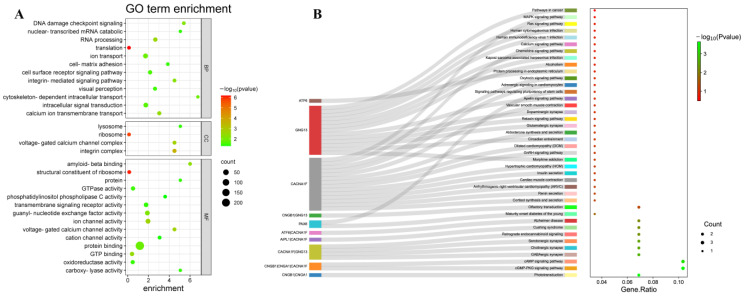
(**A**) GO classification of positively selected genes, including the top 30 significant GO categories (*p* < 0.05). BP: Biological process; CC: Cellular component; MF: Molecular function. (**B**) Sankey plots show the enrichment pathways obtained by KEGG for some of the positively selected genes associated with drought adaptation in *V. corsac*, and dot plots show the ratio between positively selected genes and the total number of genes in each enrichment pathway (*p* ≤ 0.05).

In the analysis of the positive selection of genes, we identified 1328 positively selected genes in the Corsac fox genome by comparison with the 11 species noted above, using the branch-site model in PAML (Table S22). Through enrichment analysis of these genes, we obtained 30 significantly enriched GO terms (corrected *p* < 0.05) and 30 KEGG pathways (corrected *p* < 0.05). A large proportion of the significantly enriched GO terms and KEGG pathways were closely related to energy metabolism and protein reactions (Tables S23 and S24, Figure 4A). Each of the three *Vulpes* species was used as a foreground branch to screen for their positively selected genes. More functional pathways related to energy metabolism, protein response, and stress response were present in the Corsac fox compared to the Arctic fox and Red fox (Tables S25 and S26).

Among the total number of positively selected genes, 16 genes (*BBS9*, *GPR143*, *IMPG2*, *AIPL1*, *CNGA1*, *CNGB1*, *UNC119*, *CABP4*, *CACNA1F*, *BBS10*, *PAX6*, *ARR3*, *OPN1LW*, *LRIT3*, *ATF6*, and *CNNM4*) were closely associated with visual perception (GO:0007601, *p* = 0.0331262), while four genes (*PKD2L1*, *GNG13*, *TRPM5*, and *Tas2r38*) closely related to taste transduction (ko04742, *p* = 0.018621). We also identified the gene families for FACIT collagen, with nine associated genes (*COL15A1*, *COL2A1*, *COL6A1*, *COL6A3*, *COL6A6*, *COL7A1*, *COL9A1*, *COL9A2*, and *COL13A1*). These genes are closely related to the PI3K-Akt signaling pathway, focal adhesion, and protein digestion and absorption (Figure 4B).

## 3. Discussion

We took advantage of Illumina short-reads and ONT long-reads in combination with Hi-C to produce a chromosome-level assembly of the Corsac fox with high contiguity, completeness, and accuracy. Theia high-quality chromosome-level assembly enabled us to study patterns of genomic variation, differentiation, and other genomic features (number and size of genes, repetitive elements, chromosomes) and to characterize regions of the genome that may be relevant to desert adaptation in the species. To better understand the Corsac fox genome assembly, basic genomic information was compared with that of two other *Vulpes* species [23,27]. Among the three species, the Corsac fox genome had the largest contig N50 (Table 1).

The tree topology obtained for the phylogenetic relationships between foxes was consistent with that of earlier studies, although there were differences in the timing of differentiation. The Red and Corsac fox are closely related, diverging from a common ancestor approximately 3.7 MYA before the present. The time of differentiation between the Corsac and Red fox was longer than that estimated based on mitochondrial genomic and nuclear DNA estimates [28,29,30,31]. This difference may be due to differences in technology. We also found that the divergence of the Arctic and Red fox occurred at 4.3 MYA, which is much more recent than the time estimated by Peng et al. based on the Arctic fox genome [23].

The Arctic fox is a circumpolar inhabitant that lives in the Arctic; the Corsac fox is mainly found in the arid and semiarid regions of central Eurasia, while the Red fox lives mainly in most of Eurasia and North Africa [11,23]. The three fox species show different adaptive traits. The gene families associated with protein responses are differentially altered in Corsac foxes living in hot environments compared to Red foxes and Arctic foxes. For both the Corsac fox and the Arctic fox, which also live in extreme conditions, it is important to use energy efficiently to cope with adverse temperatures and food shortages. Changes in the size of gene families associated with energy and material metabolism and stress reaction may help them adapt to such extreme environments. The Red fox is subject to fewer adverse effects and has easier access to food and a suitable ecological environment. The gene family associated with energy metabolism in the Red fox genome is in a contracted state. Although it is intuitive that gene family contraction is often maladaptive, it may provide an evolutionary mechanism for phenotypic adaptation [26]. This may be related to the ability of the Red fox to adapt to a wide range of ecological niches.

Protein synthesis and metabolism: Analysis of gene families showed that genes associated with physiological processes, such as protein synthesis and response, were affected by natural selection. Proteins are mainly structural and functional substances [32,33]. The main function of ribosomes is to convert the genetic code into amino acid sequences and build protein polymers from amino acid monomers, which are involved in the biological processes of translation and protein folding [34,35]. The Corsac fox genes are heavily enriched for ribosome-related gene ontology terms, including “structural constituent of ribosome,” “ribosome,” and “mitochondrial ribosome.” We also identified the biological processes associated with ubiquitin proteins, whose main function is to label proteins that need to be broken down and to act as regulators of protein interactions [36]. In the expansion of the gene family, “Hsp70 protein binding” and “heat shock protein binding” were commonly enriched gene ontology terms. Heat shock protein 70 (Hsp70) is a powerful chaperone whose expression is induced in response to a wide variety of physiological and environmental impacts, including anticancer chemotherapy, thus allowing cells to survive under lethal conditions [37,38,39,40]. Heat stress, which occurs in the cells of organisms exposed to high temperatures and water deprivation, affects transcriptional and translational processes, increasing the chances of DNA breakage and protein oxidation, ultimately leading to apoptosis or cell death [41,42]. Protein denaturation under heat stress is challenging for many organisms. Previous studies of the response of different mammals to thermal environments revealed that gene ontology terms such as “ribosome” and “translation” were most frequently enriched, which is also consistent with our findings [43]. These analyses suggest that genes associated with protein responses play a role in heat-stressed environments in the Corsac fox.

Energy metabolism: Sugar is one of the primary energy sources for living organisms and is a much more potent source of short-term energy in short periods than proteins and fats [44,45]. Glucose is metabolized in the body via three main pathways: glycolysis, aerobic oxidation, and the pentose phosphate pathway [46]. The expanded gene families and positively selected genes were enriched in glycolysis/gluconeogenesis, glucose metabolism, and glycolysis. Maintaining high levels of glucose metabolism provides the Corsac fox with sufficient energy to participate in a range of energy-intensive life activities such as predation. Studies in camels found that the evolution of genes related to sugar metabolism had occurred more rapidly than in cattle [3]. The adaptive evolution of sugar metabolism genes may be a key to a species’ ability to persist in extreme environments. Fat storage plays an important role in energy metabolism when food is scarce [47]. Camels living in desert areas and sheep under extreme conditions have a high capacity for fat metabolism [3,5]. Three enriched pathways related to fat metabolism were closely linked to the adaptation of the Corsac foxes to harsh environments. These genes may enhance the energy storage and production capacity of the species in desert environments.

Visual protection: Intense solar radiation is another challenge faced by Corsac foxes. One risk factor is that organisms exposed to UV radiation for long periods are at risk of developing eye diseases [3]. UV exposure was found to significantly increase the likelihood of cataract development in mice [48]. We investigated the genome of the Corsac fox for orthologous genes that may be involved in the adaptation of the Corsac fox’s eyes to solar radiation. BBS9 and BBS10 have been reported as causative genes in Bardet–Biedl syndrome, and their proteins are thought to play a role in protein trafficking and the function of photoreceptors that connect cilia to outer segments [49,50]. Mutations in the GPR143 gene cause severe ocular albinism [51]. IMPG2 plays a role in the retina, and its deletion causes functional degeneration of mouse optic rods and cone cells [52]. AIPL1, ATF6, and CNNM4 function in human and mouse retinal cone cells [53,54,55]. CNGA1, CNGB1, CACNA1F, PAX6, OPN1LW, and LRIT3 function in the retina and are associated with retina-related diseases [56,57,58,59,60]. The ARR3 gene is interlinked with the X chromosome and is associated with high myopia in humans [61]. UNC119 and CABP4 interact with photoreceptor synapses [62]. The gene family associated with phototransduction is in a contracted state, and prolonged exposure to intense UV light may have induced adaptive features in the photoreceptors. We suggest that these genes play a role in visual conservation in the Corsac fox and thus should be used as candidates to validate the genetic basis of vision in this species.

Dietary habits: The Corsac fox is not a specialist predator among mammals. Rodents constitute its main food source, but it has opportunistic habits and will take insects and seeds if food supplies are low [11]. We identified positively selected genes in its genome that mediate taste receptors and gustatory transmission [63], which is also reported in the omnivorous raccoon dog (*Nytereutes procyonoides*) and not reported as a common trait in canids. Moreover, a contracted gene family associated with olfactory receptors was found in the genome of the Corsac fox. We speculate that this is the genetic basis for the gradual adaptation of the Corsac fox diet to the harsh environment. Bitter-taste perception (related to the ability to taste benzothiamine) is mediated by the TAS2R38 gene [64,65]. The acid taste receptor PKD2L1 expresses proteins that accumulate in the gustatory region, where taste chemicals are detected [66]. TRPM5 and GNG13 are essential for odor transmission, with the TRPM5 gene playing a role in the transmission of bitter, fresh, and sweet tastes [67,68]. Bitter-taste receptors play an important role in avoiding toxic food consumption in the Corsac fox. We identified substance metabolism and degradation-related KEGG pathways (phosphonate and phosphinate metabolism) in the gene enrichment specific to the Corsac fox, with phosphorus being the most enriched in plant seeds. We suggest that these pathways and genes will help the Corsac fox adapt to a wide range of dietary strategies.

Stress reaction: The primary physiological response to environmental changes is cellular stress, which is counteracted by a range of cellular physiological responses [69]. To study the adaptation of the Corsac fox to drought and heat stress, we analyzed the genes involved in stress response [70]. Categories associated with DNA damage and repair showed strong positive selection in the Corsac fox genome. Organisms are chronically exposed to DNA damage factors that affect normal physiological functions, and repair mechanisms ensure overall survival by protecting DNA [71]. Genes related to oxidative stress were enriched in the expanded gene family. Accelerated evolution of related genes was also reported in the llama genome compared to that in cattle and alpacas. Our results were consistent with the finding that accelerated evolution of related genes was also reported in the llama genome compared to that in cattle and alpacas. The FACIT collagen family (fibril-associated collagens with interrupted helices) plays a role in maintaining the integrity of various tissues. Of the 46 genes in this family, we detected nine positively selected genes in the Corsac fox genome [72,73]. According to an earlier study, these nine genes play roles in the muscle, cartilage, eye, blood vessels, heart, and other tissues. These genes may play essential roles in the adaptation of the Corsac fox to harsh environments.

In this study, we have assembled the first chromosome-scale Corsac fox genome. This allowed us to use comparative genomics analysis to gain some insight into the molecular basis of adaptation to drought and extreme environments. Our findings will be further expanded in future research on drought adaptation as genomics technologies develop and more sequencing data become publicly available. Future functional validation analyses are needed to test extended gene families and positively selected genes to validate the genes associated with drought adaptation.

## 4. Materials and Methods

### 4.1. Sample, Library Construction, and Sequencing

An adult male *Vulpes corsac* was collected from the Hulunbuir Grassland, Inner Mongolia Autonomous Region, China, for genome sequencing. Genomic DNA was extracted from the muscle tissues using the SDS (sodium dodecyl benzene sulfonate) method. The quality and concentration of the extracted DNA were determined using 1% agarose gel electrophoresis and a Qubit fluorometer (Invitrogen, ThermoScientific, Waltham, MA, USA). For gene annotation, transcriptome sequencing was performed using several tissues of *Vulpes corsac*, including the pancreas, heart, stomach, kidney, spleen, brain, muscle, liver, and lungs. Animals in this study were handled according to the Guide for Care and Use of Laboratory Animals and in conformance with the guidelines established by the Ethics Committee for the Care and Use of Laboratory Animals of Qufu Normal University (permit number: QFNU2019-012).

First, the quality of the isolated genomic DNA was verified. A total amount of 0.2 μg DNA per sample was used as input material for the DNA library preparations. A sequencing library was generated using an NEB Next^®^ Ultra™ DNA Library Prep Kit for Illumina (NEB, BEVERLY, MA, USA) following the manufacturer’s recommendations, and index codes were added to each sample [74]. Briefly, genomic DNA samples were fragmented to a size of 350 bp using sonication. The DNA fragments were end-polished, A-tailed, and ligated using a full-length adapter for Illumina sequencing, followed by further PCR amplification. After PCR products were purified using an AMPure XP system (Beckman Coulter, California, USA), DNA concentration was measured with Qubit^®^3.0 Fluorometer (Invitrogen). Libraries were analyzed for size distribution using a 2100 Bioanalyzer (Agilent, California, USA) and quantified with real-time PCR (>2 nM). Clustering of the index-coded samples was performed on a cBot Cluster Generation System using an Illumina PE Cluster Kit (Illumina, San Diego, CA, USA) according to the manufacturer’s instructions. After cluster generation, the DNA libraries were sequenced on an Illumina platform, and 150 bp paired-end reads were generated [75].

DNA extracted from the same individual was used for long-read nanopore sequencing. According to the manufacturer’s protocol, 10 μg of *Vulpes corsac* genomic DNA was used for a 30 kb template library preparation using the Blue Pippin system. A nanopore library was prepared using a Ligation Sequencing Kit (SQK-LSK109), following the manufacturer’s instructions, and sequenced on the flow cells of a PromethION sequencer (Oxford Nanopore, Oxford, UK) [76].

### 4.2. Genome Survey

Illumina sequencing data were used for genome survey analysis [77]. K-mer-based analysis was used to estimate genome size and heterozygosity. The genome size calculated by dividing K-mer-number by depth was around 2412.09 Mbp, and the corrected genome size was 2389.14 Mbp. The genome heterozygosity rate was 0.38%, and the percentage of duplicate sequences was 53.90%. Based on the results of k-mer analysis using Soapdenovo2software, the contig N50 was 9,025 bp, and the scaffold N50 was 12,749 bp [78]. Analysis of Contig distribution and GC content led to the conclusion that the Corsac fox genome was generally complete and could be assembled using appropriate strategies.

### 4.3. RNA Extraction and Sequencing

RNA was extracted using the TRIzol Reagent (Invitrogen ThermoScientific, Waltham, MA, USA). RNA purity and integrity were analyzed using agarose gel electrophoresis, and a Nanodrop spectrophotometer was used to determine the RNA quality of each tissue [79]. After the RNA samples were tested, eukaryotic mRNA was enriched using magnetic beads containing oligo(dT). The mRNA was then broken into short fragments by adding a fragmentation buffer. One-stranded cDNA was synthesized using six-base random hexamers with mRNA as the template, followed by buffer, dNTPs, DNA polymerase I, and RNase H. Two-stranded cDNA was subsequently purified using purified double-stranded cDNA, which was first end-repaired, A-tailed, ligated to sequencing junctions, and fragmented using AMPure XP beads for fragment size selection. PCR amplification was performed, and the final library was obtained by purifying the PCR products with AMPure XP beads [80]. After library construction, initial quantification was carried out using Qubit 2.0 to dilute the library, followed by testing the insert size of the library using the 2100 Bioanalyzer. After the insert met expectations, the effective concentration of the library was accurately quantified using the Q-PCR method to ensure its quality. Different libraries were then pooled into a flow cell according to the effective concentration and target downstream data volume. The cBOT was formed into clusters and sequenced using the NovaSeq 6000 high-throughput sequencing platform (Illumina) to produce 150-bp paired-end readings. Quality control was performed on the sequencing data by taking all sequencing reads for image identification, decontamination, and de-functioning. Quality control statistics included the number of sequencing reads, data yield, sequencing error rate, Q20 content, Q30 content, and GC content.

### 4.4. De Novo Assembly and Assembly Results Assessment

After quality control, the filtered reads were used for pure third-generation assembly, and the genome was assembled using NextDenovo v2.3.1 (read_cutoff = 1k, seed_cutoff = 46,333). This was carried out in three principal steps. First, the NextCorrect module was used to correct the original data and obtain a consistent sequence (CNS sequence) after the correction [81,82]. Then, the NextGraph module was used for the genome assembly of the corrected reads to obtain the preliminary assembly of the genome. Finally, the original third- and second-generation data were successively used for genome correction using Nextpolish v1.0.5 (lgs_options = -min_read_len 1k -max_read_len 100k -max_depth 75), and the polished genome was obtained. BUSCO assessment refers to the assessment of genomic integrity at the genetic level [22]. For genome assembly evaluation, the tblastn was compared with the corresponding BUSCO database sequences to identify candidate regions, Augustus v4.1 software was used for gene structure prediction, and HMMER was used for comparison to assess its integrity. CEGMA evaluation is based on a conserved protein family set (248 core genes) that exists in a large number of eukaryotes. The approach evaluates the assembled genome and the accuracy and integrity of the core genes within this genome [83]. Using CEGMA v2.5 (default), we employed information from the core genes of six model organisms to identify candidate regions in the new genome using the tblastn, genewise, and geneid. The system uses the profile of each core gene to ensure the reliability of the final prediction of the gene structure. Sequence consistency assessments use high-quality second-generation sequencing data to assess the accuracy of third-generation sequencing data assembly results at the single-base level. BWA 0.7.8 and samtools v0.1.19 were used to detect single-base variations [84].

### 4.5. Chromosome Assembly Using Hi-C Technology

Two Hi-C libraries were prepared from the muscle tissue [85]. Following the standard protocol described earlier with certain modifications, we constructed Hi-C libraries using the original sample as the input [86]. After grinding with liquid nitrogen, the sample was cross-linked with 4% formaldehyde solution at room temperature in a vacuum for 30 min. 2.5 M glycine was added to quench the cross-linking reaction for 5 min, then the sample was put on ice for 15 min. It was then centrifuged at 2500 rpm at 4 °C for 10 min, and the pellet was washed with 500 μL PBS and then centrifuged for 5 min at 2500 rpm. The pellet was re-suspended with 20 μL of lysis buffer (1 M Tris-HCl, pH 8, 1 M NaCl, 10% CA-630, and 13 units protease inhibitor), and the supernatant was centrifuged at 5000 rpm at room temperature for 10 min. The pellet was washed twice in 100 μL ice-cold 1× NEB buffer and then centrifuged for 5 min at 5000 rpm. The nuclei were re-suspended in 100 μL NEB buffer and solubilized with dilute SDS, followed by incubation at 65 °C for 10 min. After quenching SDS with Triton X-100, overnight digestion was applied to the samples with the 4-cutter restriction enzyme MboI (400 units) at 37 °C on a rocking platform. The following steps involved marking the DNA ends with biotin-14-dCTP and blunt-end ligation of the cross-linked fragments. Proximal chromatin DNA was re-ligated using a ligation enzyme. The nuclear complexes were reverse cross-linked by incubation with proteinase K at 65 °C. DNA was purified by phenol-chloroform extraction. Biotin was removed from nonligated fragment ends using T4 DNA polymerase. The ends of the sheared fragments obtained by sonication (200–600 base pairs) were repaired using a mixture of T4 DNA polymerase, T4 polynucleotide kinase, and Klenow DNA polymerase. Biotin-labeled Hi-C samples were enriched using streptavidin C1 magnetic beads. After adding A-tails to the fragment ends and ligation with Illumina paired-end (PE) sequencing adapters, Hi-C sequencing libraries were amplified using PCR (12–14 cycles) and sequenced on an Illumina PE150 [87]. Hi-C uses special experimental techniques to obtain information on the interactions between spatially linked and physically distant DNA fragments. Different contigs or scaffolds are sorted into different chromosomes based on the fact that the probability of interactions within chromosomes is significantly higher than that between chromosomes. Contigs or scaffolds of the same chromosome are sorted and oriented based on the probability of interactions decreasing with increasing interaction distance on the same chromosome. Sequenced Hi-C data were used to mount the assembled contigs/scaffolds at the chromosomal level using AllHic version 0.9.8 [88].

### 4.6. Genome Annotation

A combined strategy based on homology alignment and de novo search to identify whole-genome repeats was applied to our repeat annotation pipeline [89]. Tandem repeats were extracted using TRF via ab initio predictions. Homolog prediction commonly uses the RepBase database employing RepeatMasker v4.0.5 software and its in-house scripts (RepeatProteinMask) with default parameters to extract repeat regions [90]. Ab initio prediction built de novo repetitive elements database using LTR_FINDER, RepeatScout, and RepeatModeler with default parameters, then all repeat sequences with lengths > 100 bp and gap ‘N’ less than 5% constituted the raw transposable element (TE) library [91]. A custom library (a combination of Repbase and our de novo TE library, which was processed using UCLUST to yield a non-redundant library) was supplied to RepeatMasker for DNA-level repeat identification. Structural annotation of the genome incorporated ab initio, homology-based, and RNA-Seq-assisted predictions and was used to annotate gene models [92]. Sequences of homologous proteins were downloaded from Ensembl, the National Center for Biotechnology Information, and other sources. Protein sequences were aligned to the genome using TBLASTN (v2.2.26; E-value ≤ 1 × 10^−5^), and then the matching proteins were aligned to the homologous genome sequences for accurate spliced alignments with GeneWise v2.4.1 software, which was used to predict gene structure contained in each protein region [93,94,95]. For gene prediction based on Ab initio, Augustus v3.2.3, GeneID v1.4, Genescan v1.0, GlimmerHMM v3.04, and SNAP (29 November 2013) were used in our automated gene Prediction pipeline for RNA-seq data [96]. Transcriptome reads assemblies were generated using Trinity v2.1.1 for the genome annotation. To optimize genome annotation, the RNA-Seq reads from different tissues were aligned to genome FASTA using TopHat v2.0.11 with default parameters to identify exon regions and splice positions [97,98]. The alignment results were then used as inputs for Cufflinks v2.2.1 with default parameters for genome-based transcript assembly. The non-redundant reference gene set was generated by merging genes predicted by the three methods with EvidenceModeler v1.1.1, using the Program to Assemble Spliced Alignment (PASA) terminal exon support and including masked transposable elements as input for gene prediction [99]. Individual families of interest were selected for further manual curation by experts. Gene functions were assigned according to the best match by aligning the protein sequences to the Swiss-Prot using BLASTP (with a threshold of E-value ≤ 1 × 10^−5^) [100]. The motifs and domains were annotated using InterProScan v4.8 by searching against publicly available databases, including ProDom, PRINTS, Pfam, SMRT, PANTHER, and PROSITE. Gene Ontology (GO) IDs for each gene were assigned according to the corresponding InterPro entries. We predicted protein function by transferring annotations from the closest BLAST hit (E-value < 10^−5^) in the SwissProt database and BLAST hit (E-value < 10^−5^) in the NR database. We also mapped the gene set to KEGG pathways and identified the best match for each gene [101]. tRNAs were predicted using the tRNAscan-SE program. As rRNAs are highly conserved, we chose related species’ rRNA sequences as references and predicted the rRNA sequences using BLAST. Other ncRNAs, including miRNAs and snRNAs, were identified by searching the Rfam database with default parameters using the internal software.

### 4.7. Comparative Genomic Analyses

For phylogenetic analysis, we used OrthoFinder v2.4.0 to retrieve *Vulpes corsac* and other assessed species (*Ailuropoda melanoleuca* GCF_002007445.2_ASM200744v3, *Canis lupus familiaris* GCF_014441545.1_ROS_Cfam_1.0, *Felis catus* GCF_018350175.1_F.catus_Fca126_mat1.0, *Homo sapiens* GCF_000001405.40_GRCh38.p14, *Mus musculus* GCF_000001635.27_GRCm39, *Mustela putorius furo* GCF_011764305.1_ASM1176430v1.1, *Nyctereutes procyonoides* GCA_905146905.1_NYPRO_anot_genome, *Panthera tigris* GCF_018350195.1_P.tigris_Pti1_mat1.1, *Ursus maritimus* GCF_017311325.1_ASM1731132v1, *Vulpes lagopus* GCF_018345385.1_ASM1834538v1, *Vulpes vulpes* GCF_003160815.1_VulVul2.2) Identifying common homologous genes, we found a total of 9793 single-copy genes across the 12 species [102]. The steps were as follows: (1) perform all-vs-all sequence BLASTP alignment using DIAMOND v2.0.13 software; (2) cluster based on the Markov Cluster Algorithm (MCL) to obtain direct homologous genomes based on the sequence alignment results; (3) construct an unrooted gene tree for each ortho-group (gene_num ≥ species_num) using FastMe v2.0 software; (4) construct an unrooted gene tree based on ortho-groups using STAG (Species Tree Inference from All Genes) to construct an unrooted gene tree; (5) use STAG v2.0 software to infer the unrooted Species Tree from ortho-groups, and then use STRIDE (Species Tree [Root Inference from Gene Duplication Events]) to assign roots to species trees by the irreversibility of gene duplication events and construct a phylogenetic tree [103].

Then, the four-fold degenerate codon sites were extracted from the single-copy orthologs to estimate the divergence time between the Corsac fox and other species. The MCMCtree program in PAML was used to perform this process. Calibration time was obtained from Timetree (http://www.timetree.org, accessed on 10 February 2023), with longer calibration time leading to more reliable divergence time estimation. These data included values for *V. lagopus* and *V. vulpes* (2.9–7.4 MYA), *M. musculus* and *H. sapiens* (81.3–91.0 MYA), *F. catus* and *P. tigris* (11.6–14.7 MYA), *U. maritimus* and *A. melanoleuca* (17.3–28.0 MYA), *C. lupus familiaris* and *N. procyonoides* (6.6–17.1 MYA), Canidae and Mustelidae (43.4–48.5 MYA), Felidae and Mustelidae (52.9–57.3 MYA), and *H. sapiens* and Felidae (91.5–97.4 MYA).

The gene families identified by OrthoFinder, of which a total of 20,070 were identified in the genomes of the 12 species, included 12,957 in the sand fox genome. We used CAFE5 to examine contracted and expanded gene families [25]. A random birth-and-death model was used to estimate the size of the gene families at the ancestral nodes. Explicit modeling of rate variation between families was carried out using gamma distribution rate category modeling. A *p*-value < 0.05 was set as the cut-off. To obtain a better fit to the data, we used the GAMMA model, compared the Model Gamma Final Likelihood, and determined that −k = 3 was a better fit to the data by selecting the highest likelihood converging model [25]. We performed KEGG and GO enrichment analysis on the expansion gene family in the context of annotated genes to determine the functional implications of the expansion and contraction genes (*p* < 0.05).

Selection analysis was conducted based on phylogenetic relationships, and 9793 single-copy genes were identified. Positive selection at the DNA sequence level was tested by estimating the ratio of nonsynonymous (dN) to synonymous nucleotide substitutions (dS) between orthologous genes. The branch-site model of CODEML in PAML was used to search for positively selected genes, with *V. corsac* as the foreground branch and other mammalian species as background branches. Positively selected genes were present in the Corsac fox genome. We performed a likelihood ratio test (LRT) for the lnl values of each model pair and obtained *p*-values using the chi-square test.

## Figures and Tables

**Table 1:** Comparison of genome assemblies between *V. corsac* and other *Vulpes* species.

Feature	*Vulpes corsac*	*Vulpes vulpes*	*Vulpes lagopus*
Assembly level	Chromosome	Scaffold	Chromosome
Total length (bp)	2,348,292,563	2,421,568,072	2,345,550,353
Number of scaffolds	202	82,424	929
Scaffold N50 (bp)	132,204,642	12,472,085	131,537,142
Number of contigs	309	183,898	1456
Contig N50 (bp)	41,624,634	55,450	33,460,300
GC content (%)	41.24	41.3	41.28

## Data Availability

The data that support the findings in this study have been deposited into National Center for Biotechnology Information (NCBI: PRJNA858197, PRJNA880477, JAOVTI000000000). The genome annotation was submitted to Figshare. URL: https://doi.org/10.6084/m9.figshare.21483018.v1. Any additional information required to reanalyze the data reported in this paper is available from the lead contact upon request.

## Supplementary Materials

The following supporting information can be downloaded at: https://www.mdpi.com/article/10.3390/ijms24119599/s1.
