# Supplementary material for: High-Quality Chromosome-Level Genome Assembly of the Corsac Fox (Vulpes corsac) Reveals Adaptation to Semiarid and Harsh Environments
Source: Int J Mol Sci. 2023 May 31;24(11):9599. doi: 10.3390/ijms24119599 (PMC10253726; doi:10.3390/ijms24119599)
Supplement: Supplementary file 1 [file ijms-24-09599-s001.zip › Supplementary Figures/Supplementary Figures.pdf]

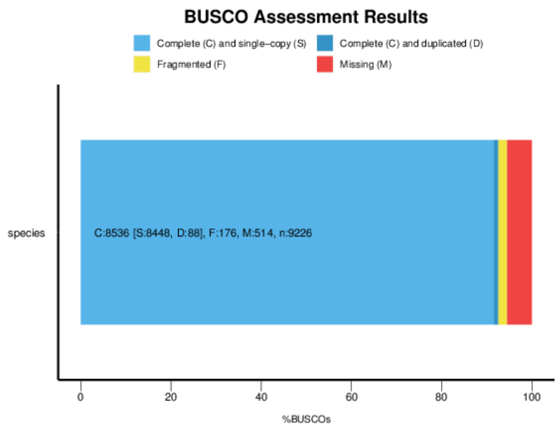

Supplementary Figure S1. Results of the Busco assessment of *V. corsac* genome

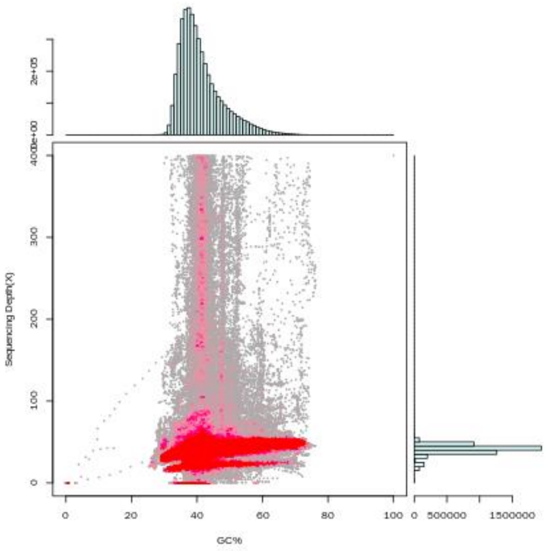

Supplementary Figure S2. The GC Depth distribution of the genome, where the abscissa is the GC content, and the ordinate is the Depth. These two values are counted sequentially in a 10Kb window.

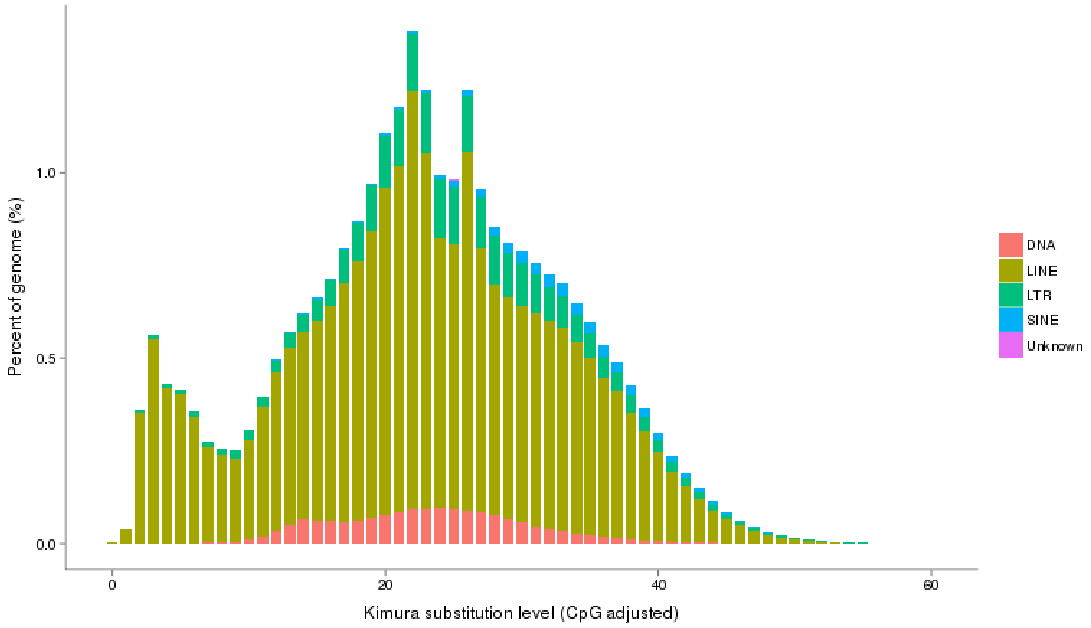

Supplementary Figure S3. RepeatableModeler transposon differentiation rate. The abscissa is the divergence degree between annotated TE sequences in the genome and corresponding sequences in the total library file, and the ordinate is the percentage of TE sequences under the divergence degree in the genome.

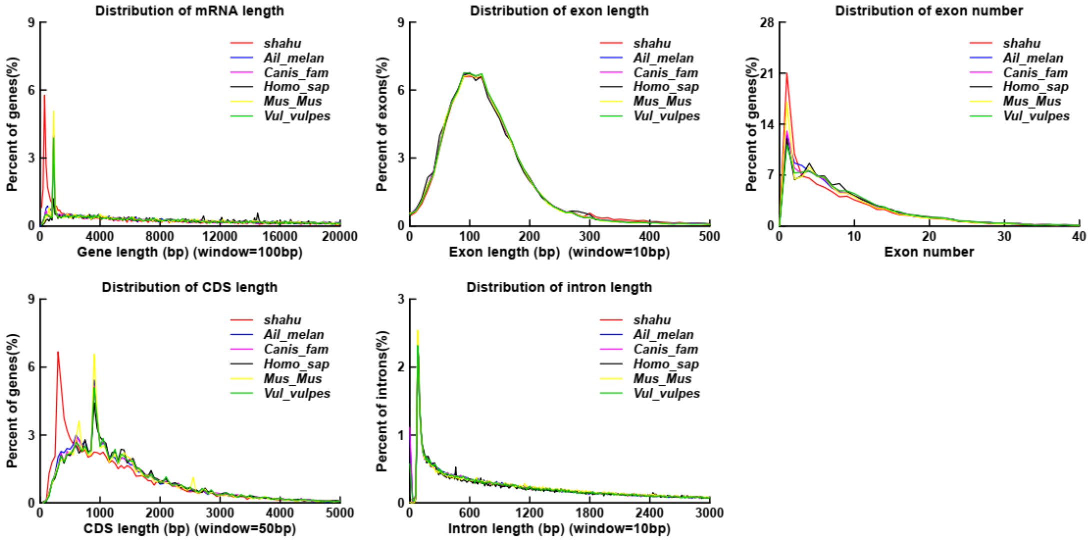

Supplementary Figure S4. Comparisons of the distribution of gene, coding sequences (CDS), exon and intron length for protein-coding genes in the genomes of *Vulpes corsac* and other species.

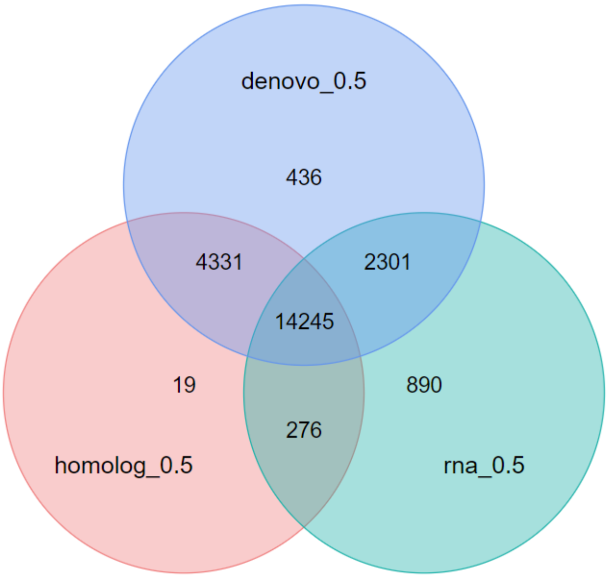

Supplementary Figure S5. Gene set evidence supporting statistics.

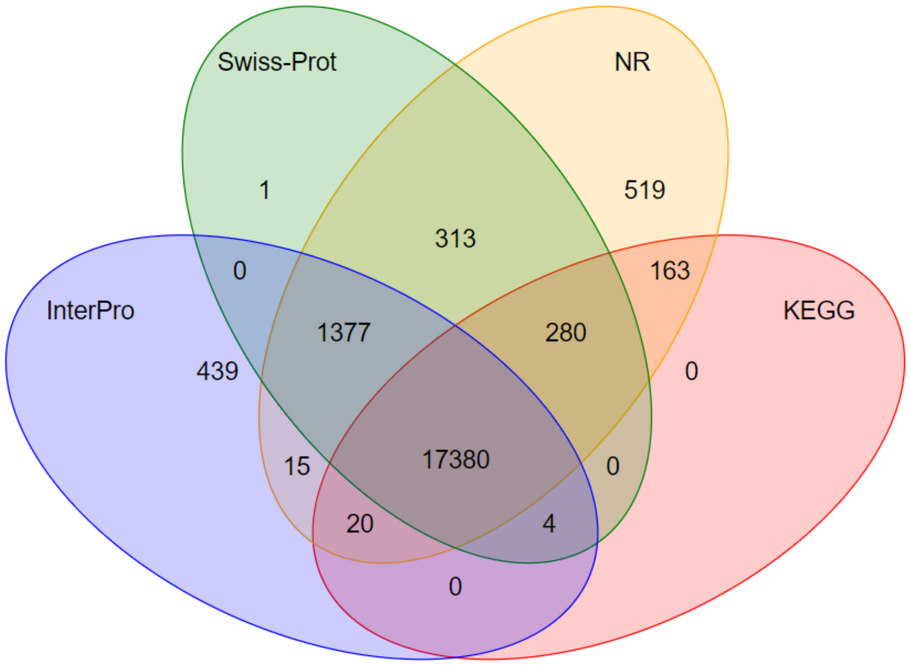

Supplementary Figure S6. The overlapping statistical mapping of Venn plot based on the gene function annotation results.

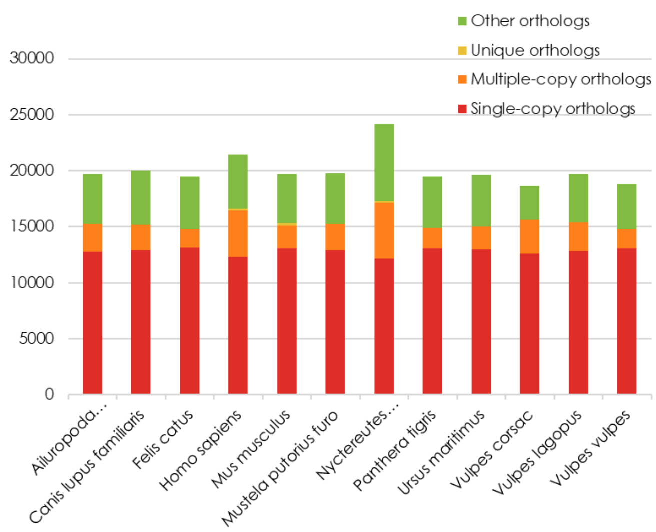

Supplementary Figure S7. Statistics of gene families among the 12 species. Single-copy and multiple-copy homologues are the families in which each species has only one or more than one copy, respectively. Unique homologues show the species--specific families. Other homologues are the genes that have matches in the other species but cannot be placed into the other orthologue categories. Unclustered genes are the genes in each species that cannot be associated with gene predictions in any of the other lineages.
