# Supplementary material for: High-Quality Chromosome-Level Genome Assembly of the Corsac Fox (Vulpes corsac) Reveals Adaptation to Semiarid and Harsh Environments
Source: Int J Mol Sci. 2023 May 31;24(11):9599. doi: 10.3390/ijms24119599 (PMC10253726; doi:10.3390/ijms24119599)
Supplement: Supplementary file 1 [file ijms-24-09599-s001.zip › Supplementary Tables/Supplementary Tables.pdf]

Supplementary Table S1. Information statistic of ONT sequencing platform

Summary statistics	
**General summary**	
Active channels:	2863
Mean read length:	11077.0
Mean read quality:	10.6
Median read length:	8749.0
Median read quality:	11.3
Number of reads:	25500341
Read length N50:	18529
Total bases:	282466915889
**Number, percentage and megabases of reads above quality cutoffs**	
>Q5:	23420644 (91.8%) 268454.7Mb
>Q7:	21765671 (85.4%) 253711.3Mb
>Q10:	16869575 (66.2%) 199523.1Mb
>Q12:	10007045 (39.2%) 123561.3Mb
>Q15:	399204 (1.6%) 4171.1Mb
**Top 5 highest mean basecall quality scores and their read lengths**	
1:	23.2 (1; f721fdcf-3c91-47f3-8aa0-ebd51bddacc7)
2:	23.1 (255; fb24517a-1b7c-4006-8bc7-27a3f6f62d28)
3:	22.1 (224; 5b0791f7-df0b-42f0-afbf-21b928ea4ef0)
4:	21.8 (207; dbb5ff61-88ef-4084-86c2-b0a28600225a)
5:	21.1 (236; 627f5aad-1409-41c2-99c8-c42cd2b9cd92)
**Top 5 longest reads and their mean basecall quality score**	
1:	1489307 (6.2; 11a0bdee-16a7-499c-85e0-4f5bbd8433ac)
2:	1258991 (3.1; 5b97f11c-8d62-4feb-9f73-c3e3b921fe51)
3:	1203819 (3.5; 85e62d04-c763-4d6a-9cec-e0b48597db51)
4:	958301 (6.0; b640dcb6-0707-46a8-a781-ac6ae2293f62)
5:	943703 (4.9; d565358a-f614-4869-b72b-494b4f7057fa)

Supplementary Table S2. Sequencing statistics generated by different platforms for *Vulpes corsac* genome assembly

Platform	Library size	Raw data (Gb)	Read length (bp)	Coverage (X)
Illumina	350 bp	132	150	55.25
Nanopore	30 kb	282	-	118.03
Hi-C	150 bp	268.65	-	112.44
Total	-	414	-	173.28

Supplementary Table S3. Statistical analysis of transcriptome sequencing results of nine organs

Sample	Raw Reads	Clean Reads	Raw Base(G)	Clean Base(G)	Q20(%)	Q30(%)	GC Content(%)
brain	22,408,035	21,601,418	6.72	6.48	97.31	92.84	50.61
spleen	23,185,049	21,930,240	6.96	6.58	97.42	93.02	49.68
lungs	21,455,208	20,798,691	6.44	6.24	97.38	92.97	51.53
heart	22,256,493	21,415,712	6.68	6.42	97.72	93.48	47.25
kidney	22,120,513	21,654,276	6.64	6.5	97.38	92.97	50.67
pancreas	21,281,017	20,663,537	6.38	6.2	97.66	93.59	56.39
muscle	21,031,287	20,430,171	6.31	6.13	97.49	93.13	52.48
liver	23,003,637	22,071,960	6.9	6.62	98.52	95.45	49.53
stomach	22,144,979	21,531,673	6.64	6.46	97.63	93.50	52.39

Supplementary Table S4. Statistics on genomic features obtained from Kmer=17 analysis

Type	Number
K-mer	17
Depth	39
n_kmer	94,071,694,805
Genome size(M)	2,412.09
Revised Genome size(M)	2,389.14
Heterozygous rate(%)	0.38
Repeat rate(%)	53.9

Supplementary Table S5. Results of the preliminary assembly of the genome of the *Vulpes corsac*

Title	Contig
Total_length	2,349,611,934
Total_number	308
Average_length	7,628,610
Max_length	113,334,919
Min_length	20,203
N50_length	47,650,574
N50_number	17
N90_length	15,609,419
N90_number	49

Supplementary Table S6. chromosome and reference genome corresponding chromosome statistical results

Sequences ID	Cluster Number	Sequences Length
Hic_asm_0	34	121,869,982
Hic_asm_1	4	79,833,746
Hic_asm_2	2	133,509,696
Hic_asm_3	3	122,329,520
Hic_asm_4	5	156,374,864
Hic_asm_5	4	135,971,213
Hic_asm_6	5	133,177,040
Hic_asm_7	3	132,204,642
Hic_asm_8	7	126,033,074
Hic_asm_9	3	122,149,359
Hic_asm_10	11	161,588,154
Hic_asm_11	7	119,000,550
Hic_asm_12	9	117,166,138
Hic_asm_13	5	112,730,960
Hic_asm_14	4	119,898,125
Hic_asm_15	8	202,442,531
Hic_asm_16	7	157,345,174
Hic_asm_17	4	64,559,422

Supplementary Table S7. Hi-c assembly result statistics.

Sample ID	Contig length	Scaffold length	Contig number	Scaffold number	Sample ID
Total	2,348,292,563	2,348,303,263	309	202	Total
Max	113,334,919	202,442,531	-	-	Max
Number>=2000	-	-	309	202	Number>=2000
N50	41,624,634	132,204,642	18	8	N50
N60	37,147,836	122,329,520	24	10	N60
N70	31,115,679	121,869,982	31	12	N70
N80	25,465,959	119,000,550	39	14	N80
N90	14,258,111	112,730,960	51	16	N90

Supplementary Table S8. Results of the BUSCO assessment of *Vulpes corsac*

Type	Number	Percent (%)
Complete BUSCOs (C)	8543	92.6%
Complete and single-copy BUSCOs (S)	8451	91.6%
Complete and duplicated BUSCOs (D)	92	1.0%
Fragmented BUSCOs (F)	175	1.9%
Missing BUSCOs (M)	507	5.5%
Total BUSCO groups searched	9226	100%

Supplementary Table S9. Results of repeat elements in *Vulpes corsac* genome.

Type	Repeat Size(bp)	% of genome
Trf	46,471,341	1.98
Repeatmasker	734,260,856	31.27
Proteinmask	210,639,551	8.97
Total	767,277,049	32.67

Supplementary Table S10. The comparison of the gene models annotated from *Vulpes corsac* genome and other mammals.

Species	Number	Average transcript length(bp)	Average CDS length(bp)	Average exons per gene	Average exon length(bp)	Average intron length(bp)
V. corsac	22,501	36,676.55	1,484.53	8.44	175.90	4,730.41
V. vulpes	19,353	41,750.63	1,706.12	9.85	173.20	4,524.49
A. melanoleuca	20,838	41,586.98	1,639.78	9.36	175.26	4,780.53
H. sapiens	23,249	4,618,0.03	1,717.97	9.68	177.52	5,123.86
M. musculus	22,173	38,263.70	1,653.48	9.08	182.13	4,531.65
C. familiaris	20,950	42,628.71	1,683.14	9.54	176.48	4,796.23

Supplementary Table S11. The statistics of gene models of protein-coding genes annotated in *Vulpes corsac*.

	Gene set	Number	Average transcript length(bp)	Average CDS length(bp)	Average exons per gene	Average exon length(bp)	Average intron length(bp)
De novo	Augustus	29,749	21,107.93	1,096.74	5.91	185.51	4,073.89
	GlimmerHMM	556,861	3,661.82	440.55	2.40	183.88	2,307.68
	SNAP	136,166	29,627.45	737.40	4.68	157.68	7,857.69
	Geneid	35,334	35,203.88	1,107.33	5.99	184.97	6,837.77
	Genscan	50,391	32,950.69	1,238.42	7.43	166.68	4,931.88
Homolog	Amel	25,017	21,935.94	1,294.92	6.98	185.50	3,451.23
	Cfam	75,912	6,078.28	480.44	2.75	174.92	3,204.94
	Hsap	24,313	22,426.79	1,329.14	7.02	189.25	3,502.64
	Mmus	23,039	23,123.71	1,373.25	7.21	190.40	3,501.03
	Vvul	38,044	14,984.97	921.78	5.08	181.55	3,449.05
RNAseq	PASA	69,156	32,082.81	1,258.34	7.50	167.76	4,741.74
	Cufflinks	65,976	43,404.43	3,227.80	8.99	359.10	5,029.29
EVM		23,974	33,327.00	1,403.90	7.95	176.69	4,596.32
Pasa-update*		23,685	35,041.44	1,440.85	8.12	177.51	4,721.04
Final set*		22,501	36,676.55	1,484.53	8.44	175.90	4,730.41

* Contains the UTR area, others do not.

Supplementary Table S12. The number of genes with homology or functional classification for *V. corsac*

	Number	Percent(%)
Total	22,501	-
Swissprot	19,355	86.00
Nr	20,067	89.20
KEGG	17,847	79.30
InterPro	19,235	85.50
GO	14,239	63.30
Pfam	17,425	77.40
Annotated	20,511	91.20
Unannotated	1,990	8.80

Supplementary Table S13. Classification of ncRNAs in *V. corsac* genome.

Type		Copy number	Average length(bp)	Total length(bp)	% of genome
miRNA		21,907	113.10	2,477,726	0.11
tRNA		16,395	80.77	1,324,287	0.056393
rRNA	rRNA	481	197.30	94,902	0.004041
	18S	37	492.46	18,221	0.000776
	28S	180	315.69	56,825	0.002420
	5.8S	10	151.10	1,511	0.000064
	5S	254	72.22	18,345	0.000781
snRNA	snRNA	2,448	117.99	288,838	0.012300
	CD-box	307	92.95	28,537	0.001215
	HACA-box	283	142.40	40,300	0.001716
	splicing	1,804	117.06	211,179	0.008993

Supplementary Table S14. 25 significantly Unique genes families of *V. corsac* GO-terms.

ID	Class	Description	P-value	Count
GO:0000276	CC	mitochondrial proton-transporting ATP synthase complex, coupling factor F(o)	0.021065874	2
GO:0000413	BP	protein peptidyl-prolyl isomerization	8.89E-15	4
GO:0000786	CC	nucleosome	0.012241502	2
GO:0003735	MF	structural constituent of ribosome	1.87E-81	33
GO:0003755	MF	peptidyl-prolyl cis-trans isomerase activity	2.43E-09	4
GO:0004298	MF	threonine-type endopeptidase activity	6.38E-06	2
GO:0004602	MF	glutathione peroxidase activity	2.56E-07	2
GO:0004984	MF	olfactory receptor activity	2.89E-28	7
GO:0005515	MF	protein binding	0.000667358	2
GO:0005839	CC	proteasome core complex	0.002090167	2
GO:0005840	CC	ribosome	6.37E-84	32
GO:0006412	BP	translation	4.50E-85	33
GO:0006457	BP	protein folding	0.008906431	3
GO:0006979	BP	response to oxidative stress	0.000236391	2
GO:0007186	BP	G protein-coupled receptor signaling pathway	0.003058447	7
GO:0008194	MF	UDP-glycosyltransferase activity	8.64E-08	2
GO:0009190	BP	cyclic nucleotide biosynthetic process	0.000236391	2
GO:0015078	MF	proton transmembrane transporter activity	0.049751929	2
GO:0015934	CC	large ribosomal subunit	1.42E-54	9
GO:0022625	CC	cytosolic large ribosomal subunit	1.00E-11	3
GO:0031625	MF	ubiquitin protein ligase binding	1.17E-05	2
GO:0046872	MF	metal ion binding	0.016286442	4
GO:0046982	MF	protein heterodimerization activity	0.001247765	3
GO:0051603	BP	proteolysis involved in cellular protein catabolic process	0.002498472	2
GO:0090522	BP	vesicle tethering involved in exocytosis	0.013901506	1

Supplementary Table S15. 13 significantly Unique genes families of *V. corsac* KEGG pathways.

ID	Description	P-value	count
ko00400	Phenylalanine, tyrosine and tryptophan biosynthesis	0.032179623	6
ko00440	Phosphonate and phosphinate metabolism	0.032179623	6
ko00460	Cyanoamino acid metabolism	0.037442138	7
ko00524	Neomycin, kanamycin and gentamicin biosynthesis	0.026888577	5
ko00541	O-Antigen nucleotide sugar biosynthesis	0.037442138	7
ko00625	Chloroalkane and chloroalkene degradation	0.026888577	5
ko00740	Riboflavin metabolism	0.032179623	6
ko00750	Vitamin B6 metabolism	0.026888577	5
ko00920	Sulfur metabolism	0.047882186	9
ko00930	Caprolactam degradation	0.032179623	6
ko00960	Tropane, piperidine and pyridine alkaloid biosynthesis	0.037442138	7
ko02024	Quorum sensing	0.047882186	9
ko03266	Virion - Herpesvirus	0.042676275	8

Supplementary Table S23. 30 significantly Positive selection of genes of *V. corsac* GO-terms.

ID	Class	Description	P-value	Count
GO:0000077	BP	DNA damage checkpoint signaling	0.00747242	4
GO:0000184	BP	nuclear-transcribed mRNA catabolic process, nonsense-mediated decay	0.038138262	3
GO:0001540	MF	amyloid-beta binding	0.004352365	4
GO:0003735	MF	structural constituent of ribosome	7.75E-07	5
GO:0003810	MF	protein-glutamine gamma-glutamyltransferase activity	0.038138262	3
GO:0003924	MF	GTPase activity	0.032926289	11
GO:0004435	MF	phosphatidylinositol phospholipase C activity	0.047229248	4
GO:0004888	MF	transmembrane signaling receptor activity	0.037502679	17
GO:0005085	MF	guanyl-nucleotide exchange factor activity	0.008266838	21
GO:0005216	MF	ion channel activity	0.002221257	25
GO:0005245	MF	voltage-gated calcium channel activity	0.001933813	6
GO:0005261	MF	cation channel activity	0.044879885	5
GO:0005515	MF	protein binding	0.013848187	242
GO:0005525	MF	GTP binding	0.003592925	11
GO:0005764	CC	lysosome	0.038138262	3
GO:0005840	CC	ribosome	1.47E-06	4
GO:0005891	CC	voltage-gated calcium channel complex	0.001933813	6
GO:0006396	BP	RNA processing	0.002759136	12
GO:0006412	BP	translation	6.47E-07	4
GO:0006811	BP	ion transport	0.014291574	25
GO:0007160	BP	cell-matrix adhesion	0.035489798	4
GO:0007166	BP	cell surface receptor signaling pathway	0.027500629	11
GO:0007229	BP	integrin-mediated signaling pathway	0.005847338	5
GO:0007601	BP	visual perception	0.033126223	7
GO:0008305	CC	integrin complex	0.000218224	8
GO:0016491	MF	oxidoreductase activity	0.035577037	9
GO:0016831	MF	carboxy-lyase activity	0.038138262	3
GO:0030705	BP	cytoskeleton-dependent intracellular transport	0.013378858	3
GO:0035556	BP	intracellular signal transduction	0.02232378	21
GO:0070588	BP	calcium ion transmembrane transport	0.003830682	9

Supplementary Table S24. 30 significantly Positive selection of genes of *V. corsac* KEGG pathways.

ID	Description	P-value	count
ko04151	PI3K-Akt signaling pathway	0.01732286	31
ko04010	MAPK signaling pathway	0.001625064	30
ko04810	Regulation of actin cytoskeleton	0.022107756	21
ko04024	cAMP signaling pathway	0.035859365	20
ko04510	Focal adhesion	0.031960181	19
ko04068	FoxO signaling pathway	0.024694268	14
ko04512	ECM-receptor interaction	0.001102759	14
ko04723	Retrograde endocannabinoid signaling	0.039568266	14
ko04142	Lysosome	0.049515247	13
ko00564	Glycerophospholipid metabolism	0.013373563	12
ko04726	Serotonergic synapse	0.015717456	12
ko04974	Protein digestion and absorption	0.008677039	12
ko04070	Phosphatidylinositol signaling system	0.031136544	11
ko05410	Hypertrophic cardiomyopathy	0.021454764	11
ko05412	Arrhythmogenic right ventricular cardiomyopathy	0.005963505	11
ko05414	Dilated cardiomyopathy	0.025016825	11
ko03008	Ribosome biogenesis in eukaryotes	0.014864506	10
ko04361	Axon regeneration	0.042358708	10
ko04727	GABAergic synapse	0.031698872	10
ko00310	Lysine degradation	0.009956304	9
ko00562	Inositol phosphate metabolism	0.037300542	9
ko04742	Taste transduction	0.018620956	9
ko00561	Glycerolipid metabolism	0.02832796	8
ko00071	Fatty acid degradation	0.011130396	7
ko04136	Autophagy - other	0.012742057	6
ko05033	Nicotine addiction	0.029063592	6
ko00515	Mannose type O-glycan biosynthesis	0.00745141	5
ko00592	alpha-Linolenic acid metabolism	0.035866337	4
ko00650	Butanoate metabolism	0.042123858	4
ko02024	Quorum sensing	0.016543543	3

Supplementary Table S25. significantly Positive selection of genes of *V. vulpes* GO-terms.

ID	Class	Description	P-value	Count
GO:0000981	MF	DNA-binding transcription factor activity, RNA polymerase II-specific	0.000180088	27
GO:0003700	MF	DNA-binding transcription factor activity	0.007496578	38
GO:0003735	MF	structural constituent of ribosome	0.003145478	3
GO:0003995	MF	acyl-CoA dehydrogenase activity	0.049448891	3
GO:0004222	MF	metalloendopeptidase activity	0.000116641	19
GO:0004930	MF	G protein-coupled receptor activity	7.07E-11	20
GO:0004984	MF	olfactory receptor activity	3.19E-13	1
GO:0005509	MF	calcium ion binding	0.030507147	47
GO:0005515	MF	protein binding	0.000771222	227
GO:0008092	MF	cytoskeletal protein binding	0.028657367	5
GO:0008237	MF	metallopeptidase activity	4.51E-06	21
GO:0008417	MF	fucosyltransferase activity	0.006885834	3
GO:0016298	MF	lipase activity	0.000482247	5
GO:0016627	MF	oxidoreductase activity, acting on the CH-CH group of donors	0.043324981	5
GO:0019904	MF	protein domain specific binding	0.034109941	3
GO:0030983	MF	mismatched DNA binding	0.034109941	3
GO:0031418	MF	L-ascorbic acid binding	0.003530554	5
GO:0035091	MF	phosphatidylinositol binding	0.014361903	10
GO:0042803	MF	protein homodimerization activity	0.03442897	4
GO:0042813	MF	Wnt-activated receptor activity	0.034109941	3
GO:0046983	MF	protein dimerization activity	0.011228013	18
GO:0070006	MF	metalloaminopeptidase activity	0.03442897	4
GO:0005840	CC	ribosome	0.009815472	3
GO:0005882	CC	intermediate filament	0.0033031	4
GO:0016020	CC	membrane	0.024052113	86
GO:0016021	CC	integral component of membrane	9.00E-07	83
GO:0001558	BP	regulation of cell growth	0.006885834	3
GO:0006298	BP	mismatch repair	0.034109941	3
GO:0006338	BP	chromatin remodeling	0.038373202	6
GO:0006355	BP	regulation of transcription, DNA-templated	0.013633534	78
GO:0006412	BP	translation	0.006956856	3
GO:0006508	BP	proteolysis	0.015010957	37
GO:0006629	BP	lipid metabolic process	0.018933935	11
GO:0007155	BP	cell adhesion	7.83E-07	32
GO:0007186	BP	G protein-coupled receptor signaling pathway	9.64E-11	23
GO:0009072	BP	aromatic amino acid family metabolic process	0.034109941	3
GO:0016055	BP	Wnt signaling pathway	0.003282345	9
GO:0030178	BP	negative regulation of Wnt signaling pathway	0.013132667	4
GO:0030198	BP	extracellular matrix organization	0.002901524	7
GO:0045104	BP	intermediate filament cytoskeleton organization	0.000482247	5

Supplementary Table S26. significantly Positive selection of genes of *V. lagopus* GO-terms.

ID	Class	Description	P-value	Count
GO:0003735	MF	structural constituent of ribosome	0.011765233	1
GO:0004672	MF	protein kinase activity	0.005090849	20
GO:0004930	MF	G protein-coupled receptor activity	0.003395364	9
GO:0005840	CC	ribosome	0.019845722	1
GO:0005911	CC	cell-cell junction	0.002117183	2
GO:0006412	BP	translation	0.016941112	1
GO:0006468	BP	protein phosphorylation	0.005698166	20
GO:0006807	BP	nitrogen compound metabolic process	0.016577176	2
GO:0007186	BP	G protein-coupled receptor signaling pathway	0.001779476	9
GO:0008484	MF	sulfuric ester hydrolase activity	0.001058699	3
GO:0016021	CC	integral component of membrane	0.028974891	30
GO:0016773	MF	phosphotransferase activity, alcohol group as acceptor	0.000691808	3
GO:0016787	MF	hydrolase activity	0.000407666	9
GO:0016811	MF	hydrolase activity, acting on carbon-nitrogen (but not peptide) bonds, in linear amides	0.007096366	2
GO:0016831	MF	carboxy-lyase activity	0.007096366	2
